# Diagnostic Implication and Clinical Relevance of Dermatomal Somatosensory Evoked Potentials in Patients with Radiculopathy: A Retrospective Study

**DOI:** 10.1155/2021/8850281

**Published:** 2021-06-01

**Authors:** Nam-Gyu Jo, Myoung-Hwan Ko, Yu Hui Won, Sung-Hee Park, Gi-Wook Kim, Jeong-Hwan Seo

**Affiliations:** ^1^Department of Physical Medicine & Rehabilitation, Jeonbuk National University Medical School, Jeonju 54907, Republic of Korea; ^2^Research Institute of Clinical Medicine of Jeonbuk National University-Biomedical Research Institute of Jeonbuk National University Hospital, Jeonju 54907, Republic of Korea

## Abstract

**Objective:**

Dermatomal somatosensory evoked potentials (DSEPs) are used to evaluate abnormalities of the somatosensory tract. There have been some studies on the diagnostic value of DSEP in radiculopathy, but it is still controversial. The purpose of our study is to evaluate the diagnostic implication and clinical relevance of DSEPs in patients with radiculopathy by comparing DSEP findings to radiculopathy symptoms and intervertebral foramen (IVF) or spinal canal stenosis in lumbar magnetic resonance imaging (MRI).

**Methods:**

This retrospective study reviewed the medical records of patients (*n* = 59) who were examined by DSEP (each L4 and L5 dermatome) and lumbar MRI. Radiculopathy symptoms and DSEPs results were compared. For the evaluation of IVF and spinal canal size, sagittal (each bilateral L4/5 and L5/S1 IVF) and axial MR images were selected at the most stenotic level. The sizes of the IVF and spinal canal were measured by the pixel counts of selected MR images. In addition, stenosis severity was morphologically graded on a 4-point scale. DSEP results were compared with the size and grade of the IVF or spinal canal stenosis.

**Results:**

DSEPs showed high sensitivity for radiculopathy symptoms. The IVF size at L4/5 and L5/S1 (pixel counts) was significantly related to either L4 or L5 dermatomal somatosensory pathway dysfunction, respectively. However, spinal stenosis (pixel counts and grade) and IVF stenosis grade were not significantly related to DSEPs.

**Conclusion:**

This paper could be helpful in the electrophysiologic diagnosis of lumbar radiculopathy.

## 1. Introduction

Dermatomal somatosensory evoked potentials (DSEPs) are used to evaluate abnormalities of the somatosensory tract, which extends from the peripheral sensory nerve to the cerebral cortex, by recording cerebral evoked responses from cutaneous stimulation of areas of known dermatomal innervation, providing a pure sensory input to any level of the spinal cord [1]. Dawson first reported evoked potentials in humans in 1947 [2]. Since then, it has been reported to be effective or helpful in identifying lesions in various damaged sites, such as peripheral neuropathies, radiculopathies, spinal cord injuries, and cerebral damage [3, 4].

Radiculopathy refers to a set of conditions caused mainly by the pinching of a nerve root in the spinal column. This can result in pain, weakness, numbness, or difficulty controlling specific muscles [5, 6]. The most common sources of radiculopathy are conditions that cause direct compression of nerve roots, including intervertebral disc herniation and degenerative spinal stenosis. [7] Through magnetic resonance imaging (MRI), the degree of nerve root compression and anatomical cause can be determined [8]. However, clinically speculated results do not necessarily match MRI findings for nerve root compression [9].

Needle electromyography (EMG) is the useful electrophysiologic method of diagnosing spinal radiculopathy. Through needle EMG, it is possible to identify sings of motor denervation such as positive sharp wave and fibrillation and to specify the compressed nerve roots [8]. However, needle EMG has some limitations, including patient discomfort due to relatively multiple needle insertions during the study and a debatable diagnostic value. The sensitivity and specificity of needle EMG for root involvement vary considerably in different studies (sensitivity of 60–79% and specificity of 40–100%) [3, 10]. Therefore, the role of DSEPs as an assistive electrodiagnostic method would be valuable.

In the diagnosis of radiculopathy, many studies have examined the diagnostic value of DSEPs [11–13]. However, conflicting opinions about the diagnostic value of DSEPs in lumbar radiculopathy are also reported, which indicate that more studies are needed to optimize its usefulness as a diagnostic procedure [4].

There have been some studies on the diagnostic value of DSEPs compared to imaging, but it is still controversial [14, 15]. The purpose of our study was to evaluate the diagnostic implication and clinical relevance of DSEPs in patients with radiculopathy by comparing DSEP findings to radiculopathy symptoms and IVF or spinal canal stenosis in lumbar MRI.

## 2. Materials and Methods

### 2.1. Study Subjects

This retrospective study was approved by the institutional ethics committee of Jeonbuk National University Hospital (Approval number: CUH 2020-03-039). We reviewed the medical records of patients (*n* = 59) who were clinically suspected to have L4 or L5 lumbar radiculopathy as they had not only low back pain but also radiating pain and numbness or weakness of the lower extremities. Inclusion criteria were patients who have radiating pain of lower extremity and were examined by DSEP and lumbar spine MRI from 2017 to 2019. Exclusion criteria were patients with myelopathy or peripheral neuropathy in nerve conduction study (NCS). The medical records of 59 patients with 236 DSEP findings (bilateral L4 and L5 dermatome) were reviewed.

### 2.2. Method

**Figure 1:**
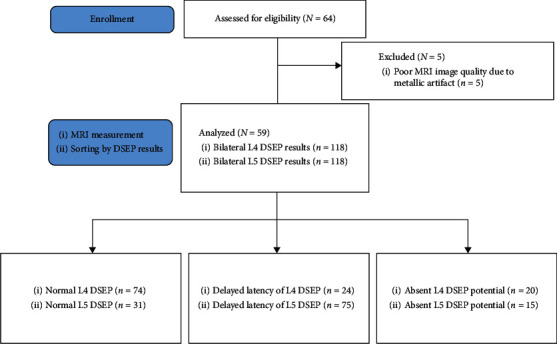
Participant flow diagram. DSEP: dermatomal somatosensory evoked potentials.

Medical records of the subjects were collected for age, sex, radiculopathy symptoms such as weakness or numbness, location of symptoms, and electrodiagnostic findings. DSEP tests were performed using a Medelec Synergy Electromyogram and Evoked Potential (EMP/EP) machine (Medelec Synergy, Oxford Instruments Medical; Surrey, United Kingdom), for which the intensity of stimuli was set at 2.5 times the sensory threshold. The sweep velocity, sensitivity, filter setting, stimulation duration time, and stimulation frequency were set at 10 ms/division, 5 uV/division, 20–1500 Hz, 0.1 ms, and 3 Hz, respectively. Encephalographic needle electrodes were used to record DSEPs. Active recording electrodes were placed on C3′ or C4′ based on the 10–20 international encephalographic system. A reference electrode was placed on Fz. It reduces the influence of subcortical far-field potentials and results in a clear cortical response compared to the noncephalic reference [16]. A ground electrode was placed between the stimulating electrode and the recording electrode. According to the suspected lesion level (L4 or L5 dermatome), a stimulating electrode was placed on the midpoint of a line between the medial malleolus and the medial epicondyle of the tibial bone for the fourth lumbar dermatome and on the lateral third of a line connecting the malleoli of the ankle for the fifth lumbar dermatome, with the use of a surface electrode. Patients were recommended to assume a relaxed posture if possible. Normal onset latency (P1-latency) is less than 47.8 ms in L4 DSEPs and 43.5 ms in L5 DSEPs. The normal side-to-side difference is less than 2.5 ms in latency [17, 18]. L4 or L5 DSEP results (*n* = 118, respectively) were divided into normal, delayed latency (delay), and absent response (absence) groups (Figure 1). Since there are many bilateral abnormal findings in our data, it is difficult to apply the side-to-side comparison for amplitude in the process of analysis, so the abnormal results were divided into delayed latency and absent response.

For the evaluation of IVF and spinal canal sizes, sagittal images to measure IVF size and axial images to measure spinal canal size were selected. Each image was selected at the narrowest bilateral L4/5 and L5/S1 IVF and the most stenotic axial level above the IVF corresponding to the DSEP level. In other words, when L4 DSEPs were analyzed, the lumbar spinal canal stenosis was measured at or above the L4/5 intervertebral disc level. When L5 DSEPs were analyzed, the lumbar spinal canal stenosis was measured at or above the L5/S1 intervertebral disc level. The size was defined by pixel counts in the sagittal view of the IVF and the axial view of the thecal sac area using the Magnetic Lasso Tool in Adobe Photoshop CC 2019 (Adobe, San Jose, CA) with reference to previous study measuring specific anatomic areas on MRI image with pixel counts [19]. The results of each MR image were compared and analyzed with the corresponding DSEP results.

IVF stenosis was graded on a 4-point scale according to morphological features on MRI. Grade 0 refers to the absence of foraminal stenosis. Grade 1 refers to mild foraminal stenosis showing perineural fat obliteration surrounding the nerve root in two opposing directions (vertical or transverse). This involves contact with the superior and inferior portions of the nerve root or anterior and posterior portions of the nerve root. No evidence of morphological change in the nerve root is shown. Grade 2 refers to moderate foraminal stenosis showing perineural fat obliteration surrounding the nerve root in four directions without morphological change in both the vertical and transverse directions. Grade 3 refers to severe foraminal stenosis showing nerve root collapse or morphological change [20]. Spinal canal stenosis was also graded on a 4-point scale according to morphological features on MRI. Grade 0 refers to no or minor stenosis showing clearly visible cerebrospinal fluid (CSF) inside the dural sac but with an inhomogeneous distribution. Grade 1 refers to moderate stenosis showing the rootlets occupying the whole dural sac but where they can still be individually distinguished. Some CSF is still present giving a grainy appearance to the sac. Grade 2 refers to severe stenosis where no rootlets can be recognized; the dural sac demonstrates a homogeneous gray signal with no visible CSF signal, as well as the presence of posterior epidural fat. Grade 3 refers to extreme stenosis in addition to no recognizable rootlets with no posterior epidural fat [21].

### 2.3. Statistical Analysis

Statistical analysis was performed using SPSS 23.0 for Windows (IBM, Armonk, NY). Data are presented as means (SDs) for continuous variables and frequencies for categorical variables. The general characteristics of the subjects were analyzed using descriptive statistics. Based on radiculopathy symptoms and DSEP results, the sensitivity, specificity, positive predictive value (PPV), and negative predictive value (NPV) were obtained. Effects of IVF stenosis and lumbar spinal canal stenosis on DSEP results were analyzed using logistic regression analysis. Intergroup differences between the normal, delay, and absence groups were analyzed using a ranked analysis of covariance (ANCOVA), with age and sex as covariates. Each ranked ANCOVA was followed by planned multiple pairwise comparisons with Bonferroni correction to *p* ≤ 0.05.

## 3. Results

### 3.1. General Subject Characteristics

This retrospective study reviewed 59 patients (29 males and 30 females; Table 1) who were examined by DSEP and lumbar spine MRI from 2017 to 2019. The mean age was 55.2 years (range: 21–85 years). Twenty-eight patients had bilateral symptoms and thirty-one patients had a unilateral symptom.

### 3.2. Comparison between Side of Radiculopathy Symptoms and DSEP Results

Radiculopathy symptoms and DSEP findings were compared in 118 legs of 59 patients. Of the 59 patients, 28 had bilateral radiculopathy symptoms and 31 had unilateral symptoms. Among the 118 legs, there were 87 symptomatic legs and 31 asymptomatic legs. A comparison of the DSEP results with radiculopathy symptoms revealed that DSEPs showed a relatively high sensitivity (93.1%), positive predictive value (82.7%), negative predictive value (70.0%), and a relatively low specificity (45.2%) (Table 2).

### 3.3. Effects of Lumbar IVF or Spinal Canal Size and Stenosis Grade on DSEP Findings

Logistic regression analysis was done for the effects of IVF stenosis and lumbar spinal canal stenosis on DSEPs. The effect of IVF stenosis (measured by pixel count) on DSEPs was significant in L4 (*p*=0.01) and L5 DSEPs (*p*=0.029). However, the effect of lumbar spinal canal stenosis (pixel count of thecal sac area) on DSEPs was not significant. Neither IVF stenosis nor spinal canal stenosis grades were associated with DSEP results (Table 3).

### 3.4. Comparison of Size and Stenosis Grade of IVF and Spinal Canal according to DSEP Findings

**Figure 2:**
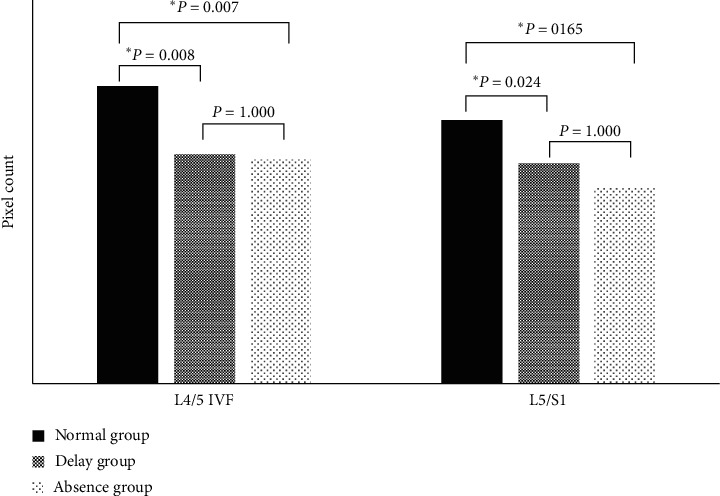
The post hoc analysis of IVF pixel counts in each group. ^*∗*^*p* < 0.05. IVF, intervertebral foramen.

Depending on the results of L4 DSEP, cases of L4/5 IVF (*n* = 118) were identified as either normal (*n* = 74), delayed latency (delay; *n* = 24), or absent (absence; *n* = 20). The mean IVF sizes were smaller in the delay and absence groups than in the normal group (mean pixel counts of normal, 237.3 ± 78.84; delay, 183.5 ± 77.94; absence, 179.4 ± 45.64). The differences between the three groups were significant (*p*=0.001). In post hoc analysis (Figure 2), there was a significant difference in pixel counts between the normal and delay groups and the normal and absence groups (*p*=0.008 in normal versus delay, *p*=0.007 in normal versus absence). However, there was no significant difference between the delay and absence groups (*p*=1.000).

The mean IVF stenosis grade was 0.78 in the normal group, 0.96 in the delay group, and 1.00 in the absence group. There was no significant difference in IVF stenosis grades between groups (Table 4).

Depending on the results of L5 DSEP, cases of L5/S1 IVF (*n* = 118) were identified as normal (*n* = 31), delayed latency (delay; *n* = 72), or absent (absence; *n* = 15). The mean IVF sizes were smaller in the delay and absence groups than in the normal group (mean pixel counts of normal, 209.7 ± 69.01; delay, 175.9 ± 76.04; absent, 158.3 ± 55.40). The differences between the three groups were significant (*p*=0.022). In post hoc analysis (Figure 2), there was a significant difference in the pixel counts between the normal and delay groups (*p*=0.024) but not between the normal and absence groups or delay and absence groups (*p*=0.165 in normal versus absence, *p*=1.000 in delay versus absence).

The mean IVF stenosis grade was 0.71 in the L5 normal group, 0.89 in the L5 delay group, and 1.13 in the L5 absence group. There was no significant difference in IVF stenosis grades between groups. Neither pixel counts nor grades as indicators of spinal canal stenosis were significantly associated with DSEP results (Table 4).

## 4. Discussion

Few studies have compared DSEPs to image studies; this is the first study comparing DSEP results and quantitative analysis of spinal stenosis through MRI. We quantitatively evaluated the degree of stenosis on MRI and analyzed the diagnostic value of DSEPs by comparing DSEP results with the degree of IVF or spinal canal stenosis by MRI and with radiculopathy symptoms. In our study, DSEPs showed a relatively high sensitivity (93.1%) and relatively low specificity (45.2%) when compared to radiculopathy symptoms, and IVF size had an effect on DSEP results. In addition, the IVF size was significantly smaller with abnormal DSEP results. However, the size or grade of spinal canal stenosis and the grade of IVF stenosis were not associated with DSEP results.

In patients with radiculopathy, electrodiagnostic studies, including needle EMG and NCS, are strongly recommended tests for the diagnosis of radiculopathy; EMG still represents the gold standard for the diagnosis of radiculopathy [22]. However, needle EMG has some limitations, such as patient discomfort, pain, and fear. It may also be difficult with patients with infectious conditions or skin lesions. Finally, needle EMG requires a skilled examiner [23]. DSEPs are used to evaluate abnormalities of the somatosensory tract, which extends from the peripheral dermatome to the cerebral cortex, and are much less invasive than needle EMG. DSEPs are performed according to a standard protocol and may be a relatively more objective method than needle EMG, which is dependent on an examiner's ability [24]. Even if radiculopathy was suspected clinically or in imaging studies, negative findings may be observed in a needle EMG study. In some studies, the sensitivity and specificity of needle EMG vary considerably (sensitivity of 60–79% and specificity of 40–100%) [3, 10]. In these circumstances, DSEPs can be a supplementary method to increase diagnostic sensitivity. Our results showed a relatively high sensitivity (93.1%) and relatively low specificity (45.2%) in comparing radiculopathy symptoms with DSEP findings. Our results showed high sensitivity and a similar specificity compared to those of needle EMG in previous studies.

Various studies have been conducted on radiculopathy and DSEPs. Some studies have shown that DSEPs have increased diagnostic sensitivity to cervical radiculopathy and showed usability as a follow-up study according to improvement [25, 26]. In a study of patients with lower back pain and radiating pain, DSEPs showed high diagnostic power as a less invasive and useful test [27, 28]. In addition, the usefulness of DSEPs has been confirmed in the diagnosis of peripheral neuropathies, like carpal tunnel syndrome [29]. DSEP monitoring has also been attempted during lumbosacral nerve root decompression as a method of intraoperative monitoring [30]. However, the diagnostic value of DSEPs is still controversial [31].

Our study showed a significant difference in IVF size between normal and delayed or absent DSEP results, but IVF stenosis grade was not significantly related to DSEP results (Tables 3 and 4). This result was probably due to the indelicate grading system of IVF size. Most grades of IVF stenosis were grade 0 or 1 on the 4-point scale. In contrast, the range of pixel counts in our study was 64–479, which assessed stenosis more sensitively. Thus, a more delicate grading system of IVF size or a more even distribution of IVF stenosis grades would have shown a significant difference in comparison with DSEP results.

Our DSEP data showed a meaningful relationship with IVF stenosis. However, no significant results were found in the relationship between spinal canal stenosis and DSEP results (Tables 3 and 4). IVF or spinal canal stenosis can be caused by intervertebral disc herniation and degenerative spinal stenosis [7]. Neural damage or compression of sensory fibers at the IVF level or spinal canal stenosis affects the transmission of somatosensory evoked potentials and increases the chance of abnormalities in a DSEP study [32], because DSEPs involve recording from the scalp and generating input at various segmental levels by stimulating cutaneous fibers along dermatomal patterns. Our study showed that IVF stenosis had more of an effect on DSEPs than spinal stenosis. L4 or L5 nerve roots were compressed by IVF stenosis or lateral recess (a region in which the nerve root passes from the thecal sac to the entrance of the foraminal zone) stenosis rather than spinal canal stenosis [33]. The lack of association between spinal canal stenosis and DSEP results is compatible with previous studies. Thus, further research is still needed to prove the relationship between spinal canal stenosis and DSEPs.

There are some limitations to this study. The first limitation is the relatively small sample size. It is likely that the smaller sample size of those with absent DSEP results reduced our statistical power (*n* = 20 in L4 DSEP level and *n* = 15 in L5 DSEP level). Second, this study did not document the signs of denervation on the EMG. Third, if DSEP is performed in the diagnostic phase without lumbosacral MRI, it may be a time-consuming approach that requires examination of many suspected lesion levels (L3, L4, L5, and S1 for each limb). The needle EMG, on the other hand, makes it possible to detect pathologic signs in the muscles pertaining to the lumbosacral roots in a shorter time.

## 5. Conclusions

Our results showed that L4/5 and L5/S1 IVF size was significantly related to L4 or L5 dermatomal somatosensory pathway dysfunction, respectively. DSEPs showed a high sensitivity for suspected lumbar radiculopathy. Thus, we suggest that DSEP studies could be helpful in the electrophysiologic diagnosis of lumbar radiculopathy.

## Figures and Tables

**Table 1:** General subject characteristics.

Subject	*N* = 59
Male/female	29/30 49.2%/50.8%)
Mean age ± SD (years)	54.7 ± 15.6
Unilateral/bilateral symptoms	28/31 (47.5%/52.5%)
Spectrum of symptoms^*∗*^	
Numbness	64
Radiating pain	28
Weakness	8

SD: standard deviation. ^*∗*^May overlap.

**Table 2:** Distribution of DSEP results according to radiculopathy symptoms.

	Abnormal DSEP findings	Normal DSEP findings	Total
Symptomatic leg (*n* = 87)	81 (true positive)	6 (false negative)	87
Asymptomatic leg (*n* = 31)	17 (false positive)	14 (true negative)	31
Total	98	20	118

DSEP: dermatomal somatosensory evoked potential.

**Table 3:** Logistic regression of lumbar IVF and spinal canal stenosis with DSEP findings.

Dependent variable	Independent variable	Odds ratio	*p*
L4 DSEP results^†^	Pixel count of IVF at L4/5 level	0.988	0.001^*∗*^
	Grade of IVF stenosis at L4/5 level	0.968	0.904
	Pixel count of thecal sac area	1.001	0.253
	Grade of spinal canal stenosis	0.780	0.471

L5 DSEP results^†^	Pixel count of IVF at L5/S1 level	0.993	0.029^*∗*^
	Grade of IVF stenosis at L5/S1 level	1.268	0.417
	Pixel count of thecal sac area	0.999	0.396
	Grade of spinal canal stenosis	0.638	0.148

^*∗*^ *p* < 0.05. ^†^Classified as normal or abnormal results. DSEP, dermatomal somatosensory evoked potentials; IVF, intervertebral foramen.

**Table 4:** Comparison of size (pixel counts) and stenosis grade of IVF and spinal canal according to DSEP findings.

	L4 DSEP				L5 DSEP			
	Normal (*n* = 74)	Delay (*n* = 24)	Absence (*n* = 20)	*p*	Normal (*n* = 31)	Delay (*n* = 75)	Absence (*n* = 15)	*p*
Pixel counts of IVF^†^	237.3 ± 78.84	183.5 ± 77.94	179.4 ± 45.64	0.001^*∗*^	209.7 ± 69.01	175.9 ± 76.04	158.3 ± 55.40	0.022^*∗*^
Grade of IVF stenosis^†^	0.78 ± 0.85	0.96 ± 0.81	1.00 ± 0.80	0.245	0.71 ± 0.82	0.89 ± 0.80	1.13 ± 0.99	0.282
Pixel counts of thecal sac area	547.4 ± 275.4	595.8 ± 344.0	586.4 ± 207.9	0.497	423.2 ± 249.7	418.2 ± 178.5	391.8 ± 159.1	0.919
Grade of spinal stenosis	1.40 ± 0.66	1.41 ± 0.71	1.35 ± 0.67	0.864	1.61 ± 0.80	1.46 ± 0.71	1.47 ± 0.74	0.627

Date are presented as mean ± SD. ^*∗*^*p* < 0.05. ^†^L4/5 level in L4 DSEP or L5/S1 level in L5 DEP. DSEP, dermatomal somatosensory evoked potentials; IVF, intervertebral foramen.

## Data Availability

The data used to support the findings of this study are available from the corresponding author upon request.
